# Supplementary material for: Wound Healing and Angiogenic Profiling of Dermal Endothelial Cells Isolated From People With Type 2 Diabetes
Source: FASEB J. 2026 Jun 12;40(12):e72013. doi: 10.1096/fj.202502874R (PMC13262748; doi:10.1096/fj.202502874R)
Supplement: Supplementary file 1 — Figure S1: Growth patterns of Healthy and T2D HDMVECs. (A) Monitoring of cell line expansion and propagation reveal unique population doublings. (B) Days to confluence of each cell type was recorded through expansion and propagation of cell lines. Data are expressed as mean ± SEM. [file FSB2-40-e72013-s001.pdf]

A

Sup Fig 1.

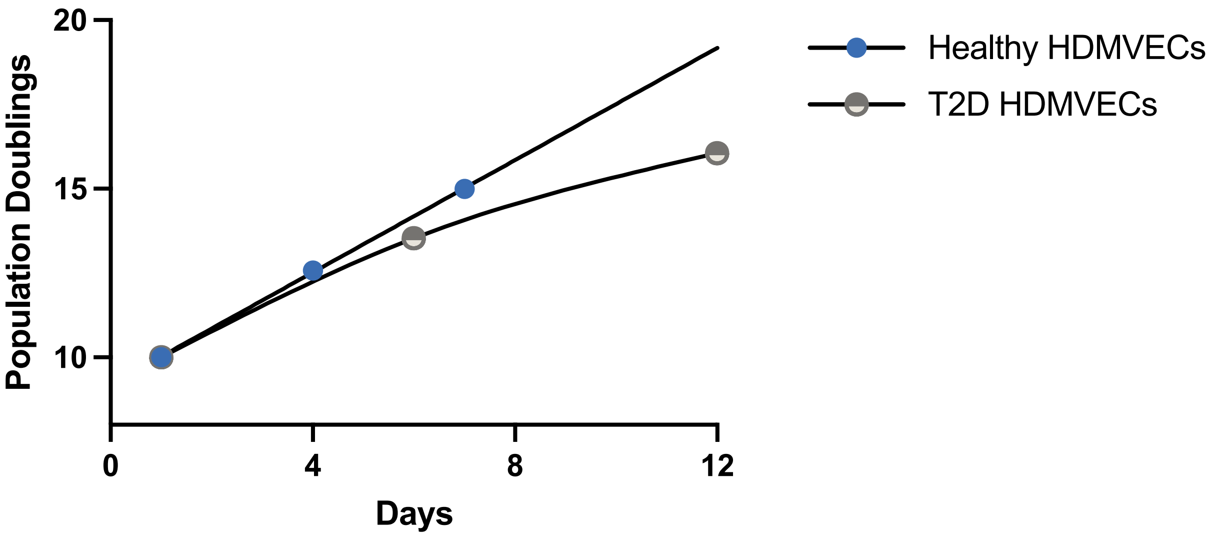

B

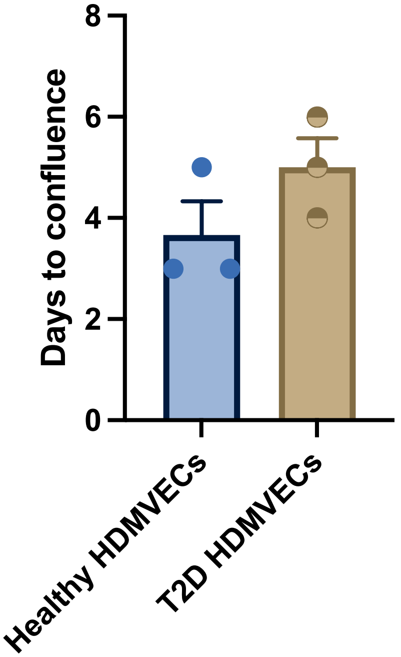

Sup Fig 1 - Growth patterns of Healthy and T2D HDMVECs. A) Monitoring of cell line expansion and propagation reveal unique population doublings. B) Days to confluence of each cell type was recorded through expansion and propagation of cell lines. Data are expressed as mean ± SEM.
