# Supplementary material for: Wound Healing and Angiogenic Profiling of Dermal Endothelial Cells Isolated From People With Type 2 Diabetes
Source: FASEB J. 2026 Jun 12;40(12):e72013. doi: 10.1096/fj.202502874R (PMC13262748; doi:10.1096/fj.202502874R)
Supplement: Supplementary file 2 — Figure S2: Timecourse analyses of LH and T2D serum treatment on functional and gene expression assays. (A) Timecourse analysis of wound healing assay. Migration index averages within conditions at 24 h. (B) Timecourse analysis of immunoblotting for eNOS. Protein expression averages across treatment times of 3 h (NT = 5; LH Serum = 5; T2D Serum = 5), 12 h (NT = 5; LH Serum = 65 T2D Serum = 5), and 24 h (NT = 6; LH Serum = 6; T2D Serum = 6) of eNOS. (C) Timecourse analysis of NOS3 and CAV1. Gene expression averages across treatment times of 3 h (NT = 6; LH Serum = 6; T2D Serum = 6), 12 h (NT = 6; LH Serum = 6; T2D Serum = 6), and 24 h (NT = 6; LH Serum = 6; T2D Serum = 6) of NOS3, and CAV1. Two‐way ANOVA was used to identify significant differences between effects. Pair‐wise comparisons were assessed via Šídák's multiple comparisons test. Data are expressed as mean ± SEM. [file FSB2-40-e72013-s002.pdf]

Sup Fig 2.

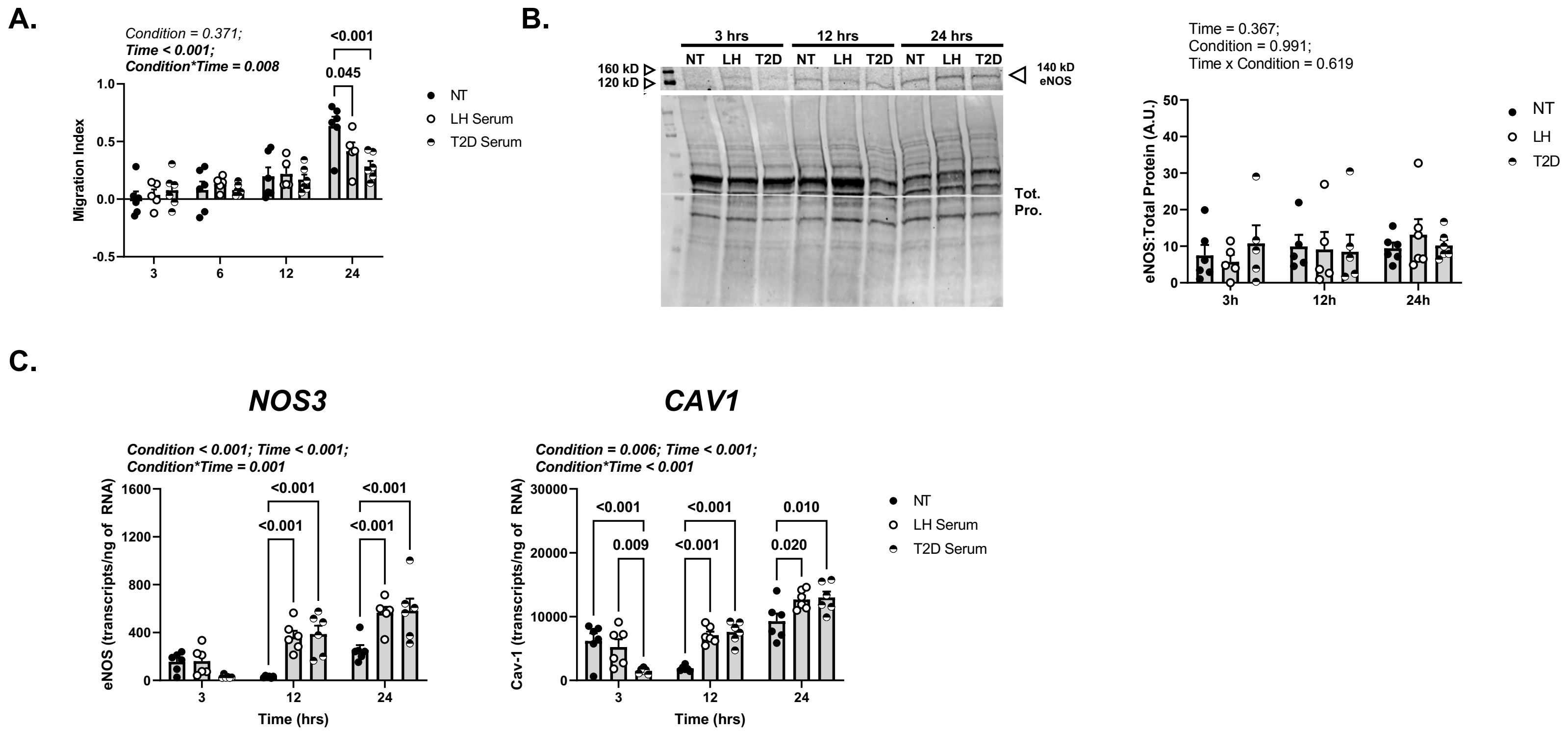

Sup Fig 2. Timecourse analyses of LH and T2D serum treatment on functional and gene expression assays. A) Timecourse analysis of wound healing assay. Migration index averages within conditions at 24 hrs. B) Timecourse analysis of immunoblotting for eNOS. Protein expression averages across treatment times of 3h (NT = 5; LH Serum = 5; T2D Serum = 5), 12h (NT = 5; LH Serum = 65 T2D Serum = 5), and 24h (NT = 6; LH Serum = 6; T2D Serum = 6) of eNOS. C) Timecourse analysis of NOS3 and CAV1. Gene expression averages across treatment times of 3h (NT = 6; LH Serum = 6; T2D Serum = 6), 12h (NT = 6; LH Serum = 6; T2D Serum = 6), and 24h (NT = 6; LH Serum = 6; T2D Serum = 6) of NOS3, and CAV1. Two-way ANOVA was used to identify significant differences between effects. Pair-wise comparisons were assessed via Šídák's multiple comparisons test. Data are expressed as mean ± SEM.
